# Anti-inflammatory response to 1,8-Cineol and associated microbial communities in Otitis media patients

**DOI:** 10.1038/s41598-024-67498-5

**Published:** 2024-07-16

**Authors:** Anke Leichtle, Mariia Lupatsii, Simon Graspeuntner, Stephanie Jeschke, Zuzana Penxová, Arwa Kurabi, Allen Frederic Ryan, Jan Rupp, Ralph Pries, Karl-Ludwig Bruchhage

**Affiliations:** 1 Department of Otorhinolaryngology, University of Luebeck, 23538 Lübeck, Germany; 2 Department of Infectious Diseases and Microbiology, University of Luebeck, Lübeck, Germany; 3 Department of Surgery/ Otolaryngology, University of California San Diego, La Jolla, USA

**Keywords:** Chronic otitis media, Inflammation, Pathogens, Microbiota, Antibiotic resistance, 1,8-Cineol, Clinical outcome, Eucalyptus oil, Microbiology, Diseases, Health care, Medical research

## Abstract

Chronic Otitis Media (COM) is defined as long term inflammation and colonization with pathogenic bacteria due to a defect or retraction of the tympanic membrane. Surgical interventions are often augmented by antibiotic resistance development and therefore, off-label treatment using the natural drug 1,8-Cineol was carried out. All COM patients underwent antibiotic therapy and middle ear surgery and developed antibiotic resistances. Microbiological investigations from the auditory canal and stool samples were performed in correlation with the clinical course. Therapy of COM patients with 1,8-Cineol revealed a clear reduction of inflammatory microbes *P. aeruginosa* and *Proteus mirabilis* in ear samples as well as intestinal *Prevotella copri*, which was associated with an improved clinical outcome in certain individuals. The present off-label study revealed manifold anti-inflammatory effects of the natural monoterpene 1,8-Cineol in Otitis media patients. A better understanding of the underlying mechanisms will improve the current treatment options and possible forms of application of this natural drug.

## Introduction

Chronic otitis media (COM) is a chronic inflammation of the middle ear and in most cases is associated with different bacterial infections of the mastoid cavity. COM is the most widespread clinical pathology that is associated with ear discharge and hearing loss^1,2^.

If there is no early intervention, ongoing inflammation results in irreversible destruction of the tympanic membrane and the surrounding middle ear structures. Hence, inflammation caused by COM is thought to alter the permeability of cochlear membranes that face the middle ear cavity, allowing bacteria and bacterial toxins to get into the inner ear^3,4^, resulting in partial of total irreversible deafness. Untreated, it may lead to otogenic complications such as mastoiditis, labyrinthitis, thrombosis of venous sinus or intracranial complications, which might end fatal^5,6^.

In everyday life, patients with COM suffer from hearing impairment, difficulties in conversations and social distancing. Without sufficient therapy, there is no way out of this descending spiral. Especially, in antimicrobial-persistent COM, middle ear surgery with complete removal of the inflammation foci and appropriate surgical rehabilitation, including reconstruction of the tympanic membrane, the middle ear ossicles or implantable hearing devices, is the only way to treat this disease^2^.

Thereby, the microbial colonization and its virulence seem to regulate infection and promote the formation of biofilms in the middle ear (ME) mucosa^7^.

Main causing pathogens are *Pseudomonas aeruginosa, Proteus mirabilis*, *Staphylococcus aureus* and *Enterobacterales*, *Candida* species and anaerobic bacteria. Similar pathogens can also be seen in advanced disease processes, e.g. mastoiditis^8,9^.

Therefore, standard treatment for COM is the administration of antibiotics to reduce the bacterial infection^10^. Pennicillins, cephalosporins and gyrase inhibitors are primarily used according to the smear test. A strict indication is the use of aminoglycosides, as it has been shown that systemic administration of aminoglycoside, a class of antibiotics against Gram-negative and Gram-postive bacteria, induce cytotoxicity in the cochlea, the vestibular system as well as the kidney^11^.

Unfortunately, inner ear sensory hair cells were shown to selectively retain aminoglycosides after systemic administration, whereas most other cell types clear the drug rather quickly. Thus, topical treatment of COM with aminoglycosides, such as neomycin, is effective, but also controversial and may be ototoxic if they enter the middle ear. This is especially relevant for children as the most vulnerable part of the population^12,13^. Potential side effects in response to antibiotic treatment always have to be considered, since moderate and high levels of multidrug-resistance especially to 3rd generation cephalosporins, Ciprofloxacin and Amoxicillin/Clavulanate are increasingly observed^14^. It is becoming more and more obvious, that, besides the local bacterial colonization, especially also the gut microbiota is closely related to the regulation of various inflammatory diseases and that bacterial metabolites and inflammatory mediators from the gut could reach distant organs via systemic circulation^15–18^. Thus, innovative treatment options are required to overcome the development of antibiotic resistances when primary and empirical treatment fails or in case of complication occurrence, to protect vulnerable patients like children and to prevent unwanted side effects.

In this study, we explored the effect of the anti-inflammatory natural and plant-based drug 1,8-Cineol on the microbiota distribution of the middle ear and intestinal bacterial colonization as well as on the clinical course of chronic Otitis media patients. The monoterpene 1,8-Cineol is a natural herbal therapeutic agent that is commonly applied to treat various chronic and acute respiratory diseases such as chronic rhinosinusitis^19^. It has been shown that 1,8-Cineol is able to significantly reduce the production of proinflammatory mediators such as TNF-α, IL-1β, and IL-6 from monocytes^20,21^ as well as the IL-4 and IL-5 production from lymphocytes^22^.

1,8-Cineol is the major bacteriostatic agent of several species of the genus eucalypt, whereas the detailed underlying mechanisms still remain unclear. Moreover, the systemic distribution of 1,8-Cineol in the human body is becoming increasingly clear. It has recently been shown, that 1,8-Cineol is present in the nasal mucosa after its oral administration for 14 days, which indicates the systemic distribution of 1,8-Cineol via the gut and the blood stream, from where it is expelled from the lungs and can unfold its anti-inflammatory effects in the respiratory tract and the mucosal tissues^23^.

However, nothing is known about the influence of anti-inflammatory natural based 1,8-Cineol on the infection process and the bacterial colonization in COM. Therefore, the aim of this study was to increase our understanding of the middle ear and intestinal microbial changes in COM patients in response to this alternative therapeutic approach in connection with the clinical course of the disease.

## Results

### Growth inhibition of inflammatory bacteria in response to 1,8-Cineol

Inhibition of bacterial growth by pure 1,8-Cineol and content of Soledum forte capsules was analyzed by disk diffusion assays using Chloramphenicol as an internal control. The COM relevant bacterial species *Staphylococcus aureus*, *Klebsiella pneumonia*, *Proteus mirabilis*, *Enterococcus faecalis*, *Enterobacter cloacae* and *Pseudomonas aeruginosa* were cultured on blood agar plates and incubated overnight prior to the start of the experiment.

**Figure 1:**
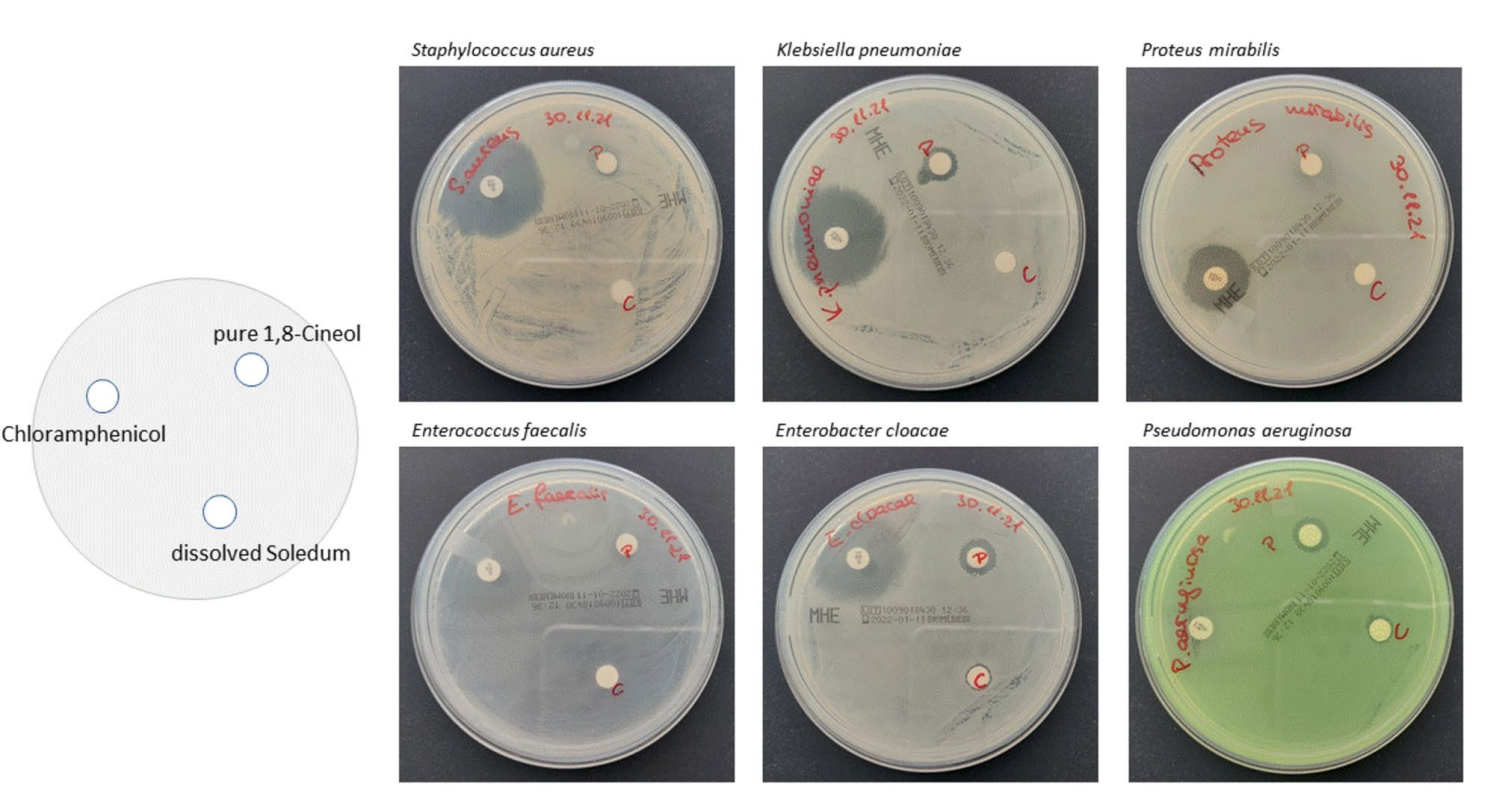
Analysis of growth inhibition of COM relevant bacterial species *Staphylococcus aureus*, *Klebsiella pneumonia*, *Proteus mirabilis*, *Enterococcus faecalis*, *Enterobacter cloocae* and *Pseudomonas aeruginosa* in response to pure 1,8-Cineol, liquid content of Soledum forte capsules and Chloramphenicol. Growth inhibition was measured in millimetres. The disks had a diameter of 6 mm.

Therefore, disks were soaked with liquid 100% 1,8-Cineol (conc. 200g/L) or the content of Soledum forte capsules (containing 200 mg 1,8-Cineol). Chloramphenicol was used as experimental internal positive control (Fig. 1).

There was an apparent growth inhibition for *Pseudomonas aeruginosa* and *Enterococcus cloacae* by the liquid from the capsules, where inhibition diameters of 8 mm could be measured in both strains (Table 1; Fig. 1). Pure 1,8-Cineol 100% clearly affected the growth of *Klebsiella pneumoniae, Enterococcus cloacae, Pseudomonas aeruginosa* and *Staphylococcus aureus* (in descending order) at small extents (Table 1; Fig. 1). There was no inhibition of *Proteus mirabilis* and *Enterococcus faecalis*, neither by pure 1,8-Cineol nor by the liquid content from the capsules. The Chloramphenicol control showed larger inhibition zones than 1,8-Cineol except for *Pseudomonas aeruginosa* (Fig. 1).

**Table 1:** Antimicrobial susceptibility testing of applied substances using disk diffusion method.

Bacteria strain	Pure 1,8-Cineol	Liquid content of Soledum capsules	Chloramphenicol
*S. aureus*	8	0	8
*K. pneumoniae*	10	0	28
*Proteus mirabilis*	0	0	18
*E. faecalis*	0	0	24
*E. cloacae*	10	8	30
*P. aeruginosa*	10	8	8

Zone of inhibition in mm.

Specific inhibition zones were measured in millimeters and are summarized in Table 1.

### Reduction of Otitis media associated bacteria in response to 1,8-Cineol

**Figure 2:**
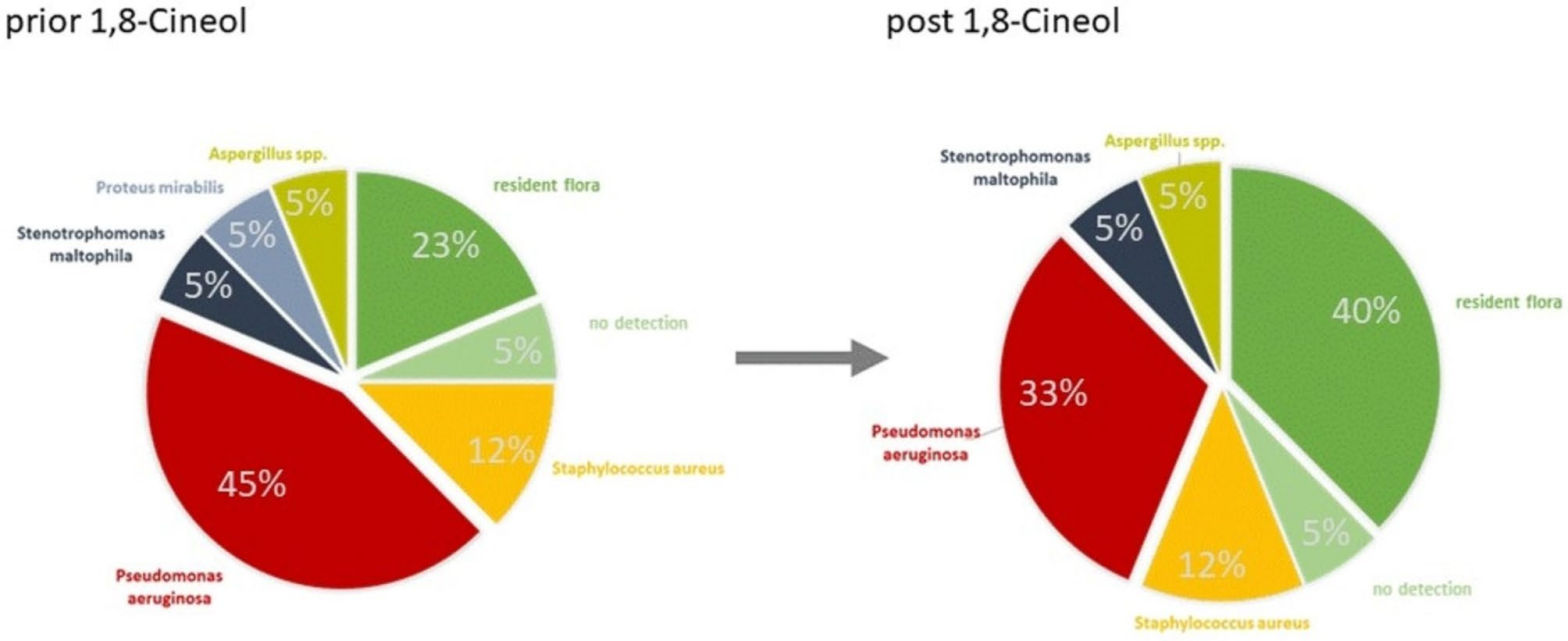
Relative abundances (in %) of different bacteria in the inner ear of Otitis media patients before and after 14 days of 1,8-Cineol administration (3 times per day 200 mg Soledum forte).

Next, swabs were taken from the auditory canal of COM patients before and after 14 days of administration of 1,8-Cineol capsules. Microbiological investigations revealed a clear redistribution of the relative abundances of the different bacterial species (Fig. 2).

Data revealed increased abundances of the so called ‘resident flora’, which means microorganisms that usually occupy a particular site of the human body. Moreover, a clear reduction of the inflammatory bacteria *Pseudomonas aeroginosa* and *Proteus mirabilis* in response to 1,8-Cineol treatment could be observed.

### Clinical course of Otitis media patients in response to 1,8-Cineol

The observed reduction of different potentially pathogenic bacteria was also reflected by an improved clinical outcome of our patient cohort, whom showed no response to the previous standard therapeutic regimen.

Evaluation of the clinical course showed a significant reduction in inflammation and a significant improvement in the overall individual situation in terms of otorrhea, pain, hearing loss, satisfaction and limitations in daily life. 1,8-Cineol was well tolerated by patients, only 20% of the treated COM patients reported on side effects such as upset stomach and ‘Cineol-breath’, however nobody reported sever symptoms and/or withdrew the therapy. All patients reported rhinosinusitis symptoms, which were also documented radiologically, but were secondary diagnosis. In addition to the documented improvement in ear symptoms, treatment with cineole also led to an improvement in rhinosinusitis symptoms, which were not in the foreground and were not perceived by the patients to be troublesome.

**Figure 3:**
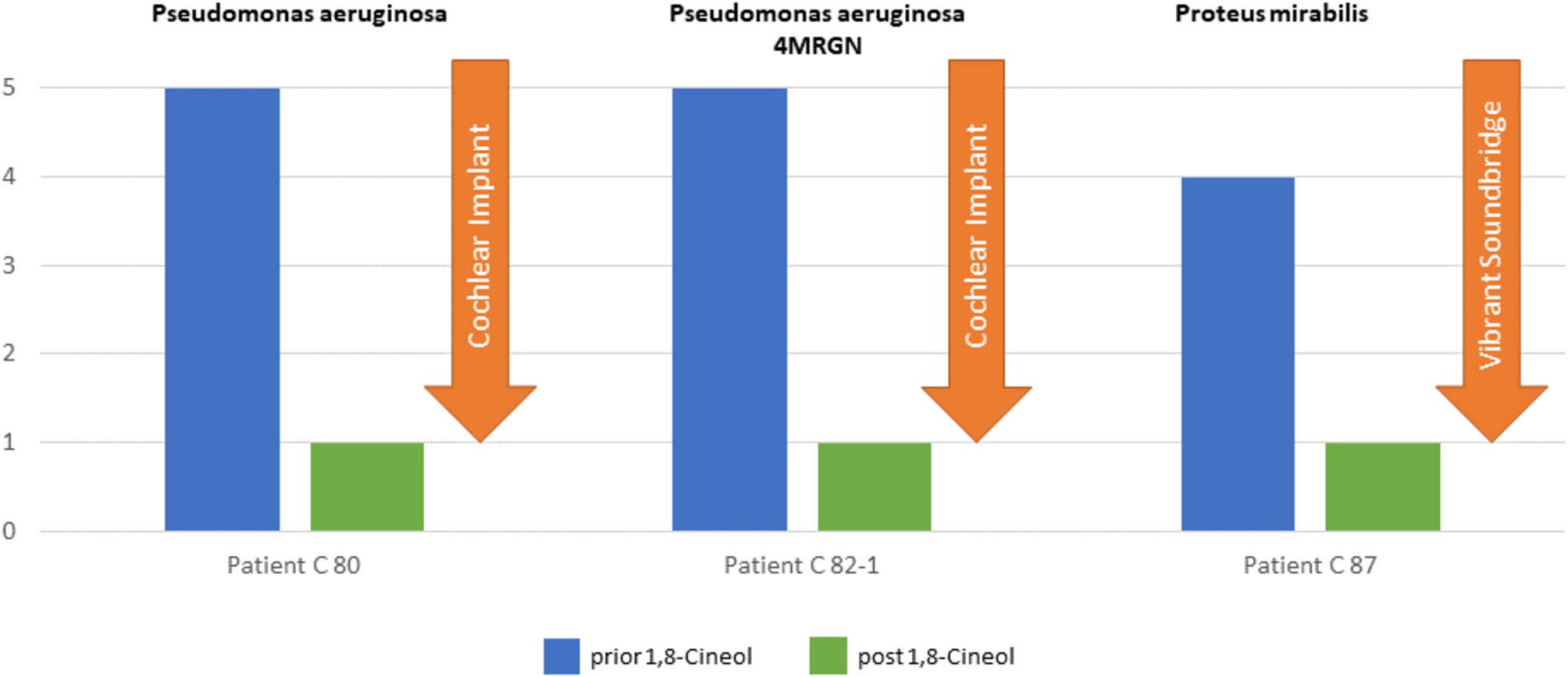
The strong reduction of *Pseudomonas aeruginosa* (patients C80 and C 82-1) and *Proteus mirabilis* (patient C 87) resulted in an improved clinical situation and enabled the surgical implantation of a cochlea implant and a vibrant soundbridge, respectively. Numbers 0 to 5 illustrate relative abundances of bacterial species (0 = undetectable; 1 = resident flora; 2 = isolated; 3 = moderate; 4 = increased, 5 = en masse).

It is particularly noteworthy that the strong reduction of inflammation associated bacteria *Pseudomonas aeruginosa* and *Proteus mirabilis* resulted in a greatly improved clinical situation and finally enabled surgical cochlea and vibrant soundbridge implantations in certain individuals (Fig. 3).

### Interplay between intestinal microbiome, treatment response status and alterations upon 1,8-Cineol administration

In addition to the microbial investigations of swabs from the auditory canal, the intestinal microbiome from Otitis media patients was analyzed based on paired fecal samples collected pretreatment and after 14 days of 1,8-Cineol oral administration. Overall, only *Ruminococcaceae* abundance was significantly increased prior to 1,8-Cineol treatment indicating minimal alterations in intestinal microbiome upon administration. After 1,8-Cineol treatment a decrease in abundance of *Prevotella copri* and *Ruminococcus gnavus* was observed, while abundances of unclassified *Bacteroides*, *Akkermansia muciniphila*, unclassified *Sutterella*, *Lachnospira* and *Enterobacteriaceae* were increased (Supplementary Fig. 1).

**Figure 4:**
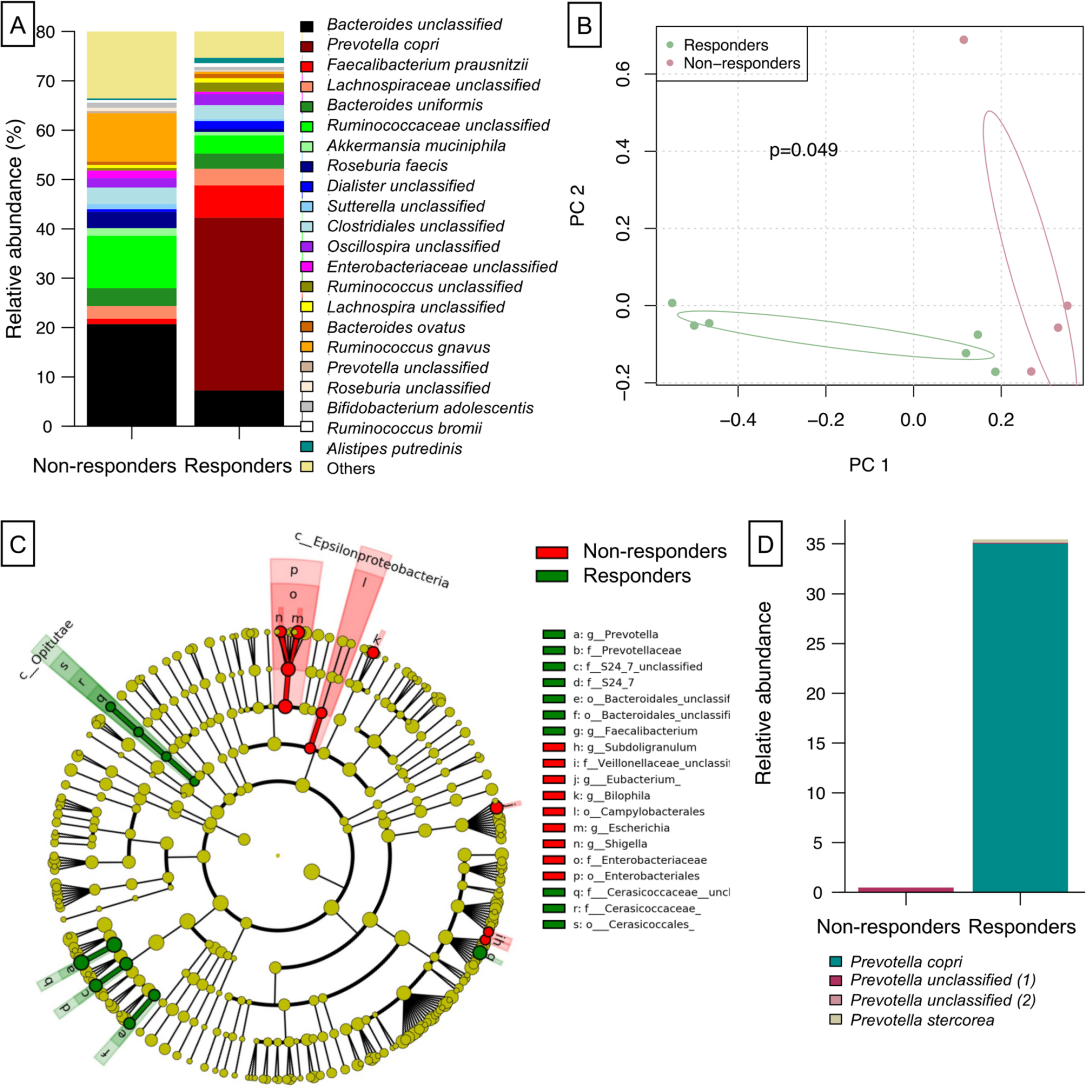
Distinctive trades of intestinal microbiome prior to treatment are associated with better responding rates to therapy with 1,8-Cineol. **A** Relative abundance comparisons between responders and non-responders groups, **B** differences in beta diversity of microbiome depending on responder’s status, **C** indicator species associated with patient group responding to therapy computed via LEfSe taxa analysis, **D** selected depiction of genus *Prevotella* species abundances in the responders and non-responders groups. Patients were defined as responders in case of none or less otorrhea and pain symptoms were presented.

To assess whether certain signatures changes in intestinal microbiota would correlate with the success of response to therapy with 1,8-Cineol, samples collected prior to herbal drug administration were grouped depending on their response status after completing the treatment. Patients presenting none to less otorrhea and decrease in pain were defined as responders. Depending on the responders status beta diversity of microbiome varied significantly between groups responders and non-responders (Fig. 4B). Comparison of microbial community composition between responders and non-responders groups revealed increase in *Prevotella copri*, *Faecalibacterium prausnitzii* and decrease in *Bacteroides* and *Ruminococcaceae* abundances (Fig. 4A). Furthermore, indicator species analysis defined genus *Prevotella* and orders *Bacteroidales* and *Cerasicoccales* as associated with responders group (Fig. 4C) with species *Prevotella copri* and *Prevotella stercorea* elevated in abundance (Fig. 4D).

## Discussion

The present off-label study evaluated the natural monoterpene medicinal 1,8-Cineol as an alternative treatment option in patients with chronic Otitis media. Our study revealed anti-microbial activities of 1,8-Cineol in vitro growth inhibition assays as well as in investigations of ear swab samples from Otitis media patients, with clear effects against commonly clinically encountered bacteria: *Klebsiella pneumoniae, Enterococcus cloacae, Pseudomonas aeruginosa, Proteus mirabilis*, and *Staphylococcus aureus*.

Antimicrobial susceptibility testing of was performed using disk diffusion method. Data revealed an apparent growth inhibition for *Pseudomonas aeruginosa* and *Enterococcus cloacae* in response to the liquid from the capsules, whereas pure 1,8-Cineol 100% clearly affected the growth of bacteria species *Klebsiella pneumoniae, Enterococcus cloacae, Pseudomonas aeruginosa* and *Staphylococcus aureus.* These observations clearly demonstrate a direct antimicrobial effect of 1,8-Cineol on certain bacteria with different sensitivities. Furthermore, no inhibition of *Proteus mirabilis* and *Enterococcus faecalis* could be detected via the disk diffusion assays, neither by pure 1,8-Cineol nor by the liquid content from the capsules. Of note, effects of 1,8-Cineol against *Proteus mirabilis* could be detected *in vivo* in Otitis media patients, which suggests as well indirect effects of 1,8-Cineol treatment, most likely via a modulated microenvironment and an activated phagocytosis activity of different immune cells in the inflammatory microenvironment of Otitis media patients. In contrast, growth inhibition of all analysed bacteria was observed in response to Chloramphenicol, which directly interferes with substrate binding in the bacterial ribosome and thus blocks the progression of growing peptides. This underlines the assumption of direct and indirect effects of 1,8-Cineol within the infectious situation of Otitis media patients and also suggests 1,8-Cineol as a promising alternative treatment option of the individual inflammatory situation.

Earlier studies have already shown anti-microbial effects of 1,8-Cineol in different inflammatory diseases^24–27^. Regarding bacterial colonization, Sokovic et al. examined anti-microbial activities of 1,8-Cineol in Minimal Inhibitory Concentration (MIC) assays and found effects especially against gram positive bacteria like *Staphylococcus aureus*. Also, gram negative bacteria like *Klebsiella pneumoniae, Proteus vulgaris* and *Pseudomonas aeruginosa* showed growth reduction in the presence of 1,8-Cineol. They also found stronger anti-microbial capacities of 1,8-Cineol compared to other compounds of the essential eucalyptus oil like α- and β-Pinene^28^.

To be noted, Ghavam et al. discovered an inhibitory effect of the essential oil of salvia on *Pseudomonas spp*., which contains mainly 1,8-Cineol and ( +)- Spathenulol^29^. *Pseudomonas spp.* are one of the main pathogens for a chronic inflammatory process in the middle ear.

Interestingly, an association between the colonization of children’s middle ears with pathogenic bacteria like *Haemophilus influenzae*, *Streptococcus pneumoniae* and *Staphylococcus aureus* and the dimensions of related hearing loss has been described during episodes of Otitis media with otorrhea. When pathogenic bacteria were detected in the children’s ears, the hearing loss was 5 dB more than in children with Otitis media but without the detection of pathogenic bacteria^30^. In addition, the groups related the uselessness of antibiotic treatment to the formation of biofilms^30^.

This effect has already been widely described in middle ear infections, especially in ears that are colonized by *Pseudomonas aeruginosa*^31,32^. 1,8-Cineol is capable to reduce the biofilm of some bacterial species and induce the death of bacterial cells^33^.

In this context, essential oils from *Eucalyptus globulus* with 1,8-Cineol as the main metabolite (65.83%) have been shown to inhibit *Streptococcus mutans* even in biofilm cultures, emulating dental plaque conditions^34^. It is most likely that biofilm formation is a major problem concerning the efficacy of anti-microbial treatment due to limited penetrability. Correspondingly, 1,8-Cineol was also able to penetrate *E. coli* biofilms, which could be enhanced under osmotic stress^35^. Vasquez et al. reported this effect in cultures with uro-pathogenic *Escherichia coli*^36^. This effect of 1,8-Cineol has also been described in otorhinolaryngological cases: Schürmann et al. described the same decrease of biofilm in chronic rhinosinusitis with *Staphylococcus aureus* as the main pathogenic bacteria^27^. Even Methicillin-resistant *Staphylococcus aureus* (*MRSA*) showed growth reduction under presence of 1,8-Cineol^33^. Hendry et al. discovered the impact of eucalyptus oil and 1,8-Cineol on the formation of biofilms by *MRSA* and *Pseudomonas aeruginosa* in the dental setting and proved that chlorhexidine-digluconate has better antibacterial effects in combination with 1,8-Cineol or eucalyptus oil than in its pure form^37^.

Investigations on pathogenic *S. aureus* revealed a growth inhibitory effect of 1,8-Cineol but also the downregulation of a protein involved specifically in biofilm formation^27^. This is a very interesting aspect, since the QS (quorum sensing) pathway is known to be required for proper formation and functioning of bacterial biofilms. QS is the regulatory communication system via chemical signals within bacterial populations and is involved in bacterial invasion, defense and distribution^38,39^. In this context, 1,8-Cineol has been shown to modulate QS related bacterial receptors^40^ as well as the cellular characteristics of the bacterial shape and size^41^. To improve the bioavailability of natural compounds such as 1,8-Cineol, the preparation of encapsulating invasomes has recently been suggested as a promising approach to efficiently treat bacterial infections^42,43^. The capsules containing 1,8-Cineol are applied orally as enteric coated capsules enabling their curative effects after their passage through the stomach within the small intestine where it spreads via the gut and the bloodstream^23^. In this sense, the first microbes encountering the substance is the gut microbial community. The gut microbiome is implicated in a wide range of diseases in remote organs and their capacity to modulate inflammation in distinct body sites has been defined to depend on nutritional aspects^43^. While there are clearly established links between gut microbiota and e.g. chronic respiratory diseases a considerable link between gut microbial communities has been suggested^18^, yet lacked clear evidence. Given the separation in patients responding to treatment by resolving COM (responders) and patients who showed generally better outcome without clearance of COM (non-responders), we sought to establish the potential of a link between the gut microbiome and the outcome of the patient´s ear health assessment. We found a drastic difference in the microbial composition of the gut between non-responders and responders.

**Figure 5:**
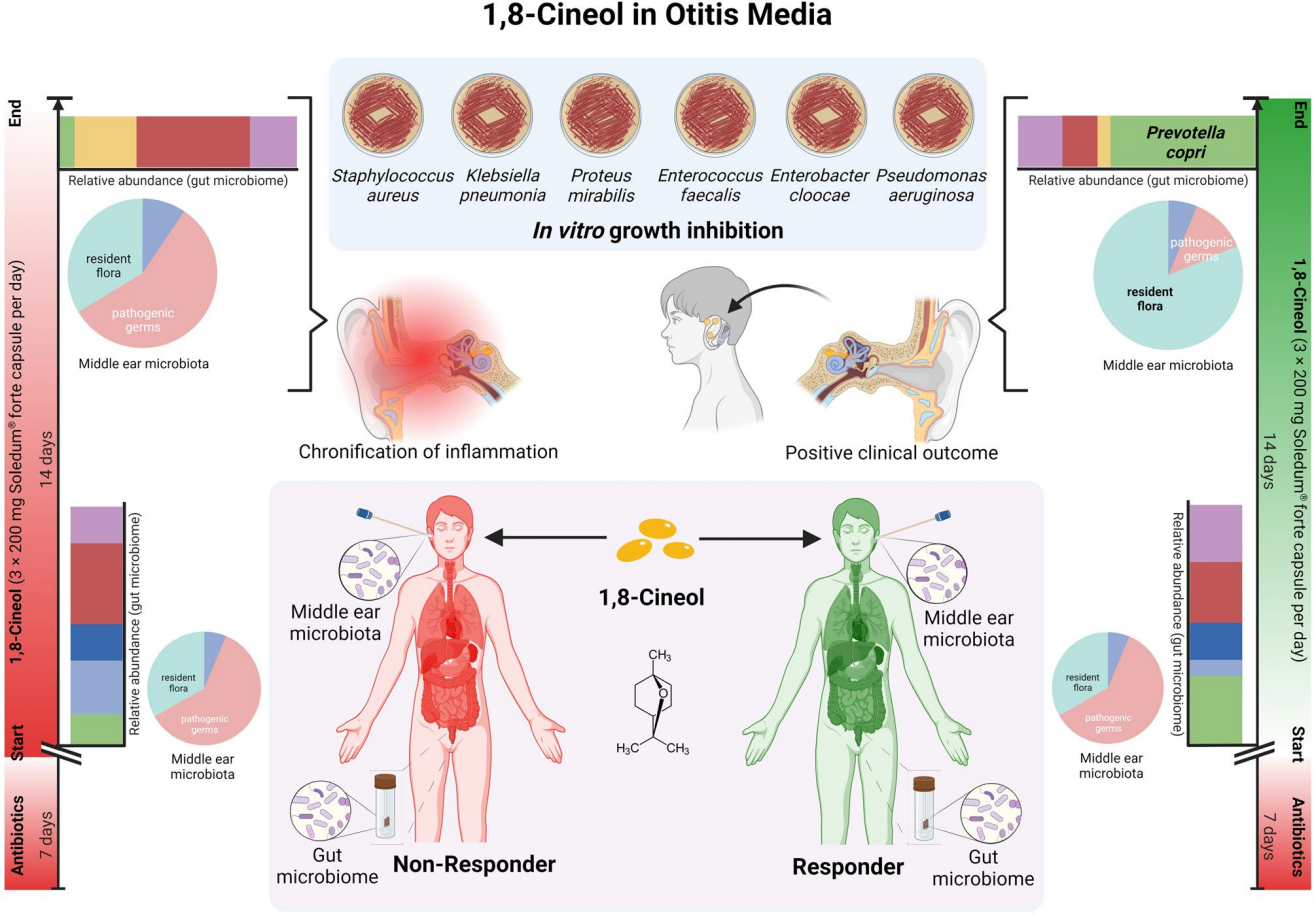
Graphical illustration of the key findings. Comparison of gut microbiota from COM patients before and after 14 days of 1,8-Cineol administration (3 times per day 200 mg Soledum forte) revealed a drastic difference in the microbial composition of the gut between non-responders and responders. Most obviously, responders revealed a high relative abundance of *Prevotella copri* with the genus *Prevotella* being significantly associated with the responder group and barely any *Prevotella* sp. being present in the gut of non-responders (created with BioRender.com, see text for details).

Most obviously, we found responders to have a high relative abundance of *Prevotella copri* with the genus *Prevotella* being significantly associated with the responder group and barely any *Prevotella* sp. being present in the gut of non-responders. It is noteworthy that a considerable abundance of *Prevotella* has been defined as the hallmark defining one of the gut enterotypes describing microbial communities in humans^44^. Figure 5 visually summarizes the key findings of our research.

In the light of the plant-originating 1,8-Cineol it may also be of particular interest that *Prevotella* is linked to fiber-enriched nutrition as known for e.g. vegetarians^45^. *Prevotella copri* has, moreover, been suggested to directly influence the metabolism of the human as a host and may act immunomodulatory e.g. via the production of branched-chained amino acids (BCAA)^46,47^.

In our data, it further appears that Bacteroides genus is increased in non-responders, which is in line with the fact that *Prevotella* and *Bacteroides* genera are negatively associated with each other^48^. We want to emphasize the fact, that *Bacteroides*-enriched enterotype Bact2 was described^49^ as promoting systemic inflammation. It can be noted that also presence of *E. coli* (as seen in the non-responders group) has been linked to worsened health outcomes in chronic diseases. Thus, the microbial composition of the gut of responders seems to have less inflammatory capacity than the microbiome of non-responders, which may play an essential role in the context of a successful COM treatment on the basis of 1,8-Cineol.

While the exact combination of actions leading to COM resolution are yet to be established, our data indicate a mixture of direct effect on the present pathogens in the middle ear and a systemic and gut microbiome axis-dependent mechanism. It appears likely, that both mechanisms need to act in conjunction when 1,8-Cineol is applied as an oral treatment in COM patients upon treatment failure with standard treatment strategies. Particular emphasis may be placed on respective microbial species present not only in the ear but also in the gut when planning COM treatment considering the absence of proinflammatory microbiota and especially the presence *Prevotella copri* as predictors for the treatment success.

An acknowledged limitation is the relatively small number of examined patients, which is due to the pilot character of our study and the fact that the therapeutic agent 1,8-Cineol is by now commonly applied to treat various chronic and acute respiratory diseases but not yet explicitly in Otitis media. In the light of required large scale cohort studies a particular emphasize will need to be given on the mechanisms that underly the potential systemic effect of *Prevotella* species upon treatment with the anti-inflammatory and anti-microbial substance 1,8-Cineol in COM.

## Methods

### Ethics statement

Medical examinations and surgical treatments were carried out at the Department of Otorhinolaryngology, University Hospital Schleswig–Holstein, Campus Luebeck. All patients have given their written informed consent. The study was approved by the local ethics committee of the University of Luebeck (approval number 22-040) and conducted in accordance with the ethical principles for medical research formulated in the WMA Declaration of Helsinki.

### Characteristics of examined patients

All enrolled patients (n = 10) in this study suffered from rhinosinusitis and persistent COM, both of which share the same respiratory epithelium (see Table S1 for further clinical details). The median age of the patients with COM was 53 years (range, 30 to 68 years) and all patients underwent antibiotic therapy and middle ear surgery and developed microbial resistances against many or all available classical antibiotics. Often, there were no therapeutic options left and therefore we treated them with 1,8-Cineol. 1,8-Cineol (CNL-1976) was used in terms of the clinically approved drug Soledum^®^ Kapseln forte (capsules) (Cassella-med GmbH & Co. KG, Cologne, Germany). For therapeutic use patients have been prescribed Soledum capsules forte (3 x 200 mg Cineol/day) over 14 days for oral administration. They did not obtain any local or systemic antibiotic treatments during this time period.

For diagnostic scoring, we examined the change of pain, hearing loss, otorrhea, pressure in the tuba auditiva and other factors related to quality of life with a questionnaire before and after off-label therapy with 1,8-Cineol administration. Furthermore, we took swabs from the auditory canal before administration and after completion of the 1,8-Cineol capsules therapy.

### In vitro growth inhibition by 1,8-Cineol

Inhibition of bacterial growth by pure 1,8-Cineol and Soledum forte was analyzed by disk diffusion assay method using chloramphenicol as an internal control. Therefore, blank disks (Oxoid^TM^, Thermo Fisher Scientific, Waltham, USA) were soaked with liquid 100% 1,8-Cineol (conc. 200g/L) or the content of Soledum forte capsules (containing 200mg 1,8-Cineol and 18 mg Sorbitol 70% solution) overnight. Chloramphenicol disks with 30 μg concentration were used as referent experimental positive control. To evaluate inhibitory degree of tested compounds COM relevant bacterial species *Staphylococcus aureus*, *Klebsiella pneumonia*, *Proteus mirabilis*, *Enterococcus faecalis*, *Enterobacter cloocae* and *Pseudomonas aeruginosa* previously isolated from patient samples (refer to Wolf et al.^50^ for our view on the importance of large-scale culturomics for clinical purposes) were cultured in sterile conditions. Overnight bacterial cultures were incubated on Columbia agar supplemented with 5 % sheep blood (bioMérieux, Marcy-l'Étoile, France) were used to prepare 0.6 McFarland standard unit suspensions in 0.85 % NaCl medium (bioMérieux, Marcy-l'Étoile, France). Thereafter 100 μl of microbial culture were plated on Mueller Hinton agar (bioMérieux, Marcy-l'Étoile, France) and the disks with cineol and antibiotic applied. Inhibitory activity was evaluated after incubating overnight based on the zone of inhibition measurements, in triplicate plates.

### Analysis of ear swabs for microbial colonization

Swabs samples were collected from the auditory canal of the COM patients by insertion of a sterile flexible cotton-wool swab (Copan eNat Swap; Mast Diagnostik GmbH, Reinfeld, Germany). Swabs were stored in sterile Skim-Milk-Tryptone-Glucose-Glycerol-Broth and later transferred to our local microbiology laboratory, as previously described^51^. The swabs were investigated for conventional bacterial culture of commensal and pathogenic bacteria of the ear. According to the standard operating procedures, the samples were plated on blood and chocolate agar plates and incubated for up to 48 h. The numbers of swabs from the external auditory canal assessed for commensal and pathogenic bacteria, were analyzed and displayed in percentages.

### Gut microbiome analysis

Analysis of the intestinal microbiota community was carried out on 10 sample pairs corresponding to pre- and post- 1,8 Cineol treatment of the patients.

The patient’s stool samples were collected in Stool Collection Tubes with DNA Stabilizer (Invitek Molecular GmbH, Berlin, Germany) and were processed using PSP® Spin Stool DNA Plus Kit (Invitek Molecular GmbH, Berlin, Germany).

The isolated DNA was amplified via targeting V3/V4 hypervariable region of 16S rRNA gene using customized primers^52^. The cycling parameters were as follows: initial denaturation at 98 °C for 30 seconds followed by 30 cycles of 98 °C for 9 seconds, 55 °C for 60 seconds and 72 °C for 90 seconds, and final extension for 10 minutes at 72 °C. Thereafter amplicon concentration was measured based on band intensity in 2 % agarose gel electrophoresis determined against the reference band in GeneRuler 100 bp DNA Ladder (Thermo Fischer Scientific, Waltham, USA). Equimolar concentrations of amplicons were pooled and after running on a gel, bands were purified via MinElute® Gel Extraction Kit (Qiagen GmbH, Hilden, Germany), final library concentration was estimated using NEBNext® Library Quantification Kit for Illumina® (New England Biolabs, Ipswich, Massachusetts, USA). Next generation sequencing was conducted using MiSeq® Reagent Kit v3 (600 cycles) (Illumina®, San Diego, California, USA), all negative controls from DNA isolation were sequenced with other samples to ensure lack of contamination and PhiX Control Library v3 (Illumina®, San Diego, California, USA) was used to control for sequencing quality, cluster generation and alignment. Raw sequences were processed via mothur 1.44.1^53^ with alignment against SILVA reference database^54^ and taxonomic assignment based on Greengenes Database^55^. Chimeric sequences were filtered using VSEARCH algorithm^56^, operation taxonomic unit allocation was performed with cutoff level of 0.03. Microbial community analysis was carried out using R (version 4.0.1), relative abundance computations were performed via graphics package^6^, differences in abundance were compared via pairwise Wilcoxon rank-sum test with corrections for false detection rate in stats package^7^. Further insights into community differences were assessed based on definition of responder group with patients who presented none or less otorrhea and pain symptoms. Beta diversity was analyzed via principle coordinates analysis using labdsv package^8^, degree of dissimilarity was computed through permutational multivariate analysis of variance using distance matrices (vegan package)^9^. Identification of indicator species was performed using Linear Discriminant Analysis Effect Size (LEfSe) via Galaxy Project Platform^57,58^.

### Informed consent

Informed consent was obtained from all subjects involved in the study.

### Supplementary Information

Supplementary Information.

## Data Availability

The data presented in this study are available on request from the corresponding author. Raw sequences used for microbiome analysis within this study are made available at the European Nucleotide Archive under accession number PRJEB71624.

## References

[1] Rovers MM, Schilder AG, Zielhuis GA, Rosenfeld RM (2004). Otitis media. Lancet.

[2] Bruchhage KL, Lupatsii M, Mollenkolk F, Leffers D, Kurabi A, Jurgens T, Graspeuntner S, Hollfelder D, Leichtle A (2023). Hearing rehabilitation and microbial shift after middle ear surgery with Vibrant Soundbridge in patients with chronic otitis media. Eur. Arch. oto-rhino-laryngol. Off. J. Eur. Fed. Oto-Rhino-Laryngol. Soc..

[3] Shi X (2016). Pathophysiology of the cochlear intrastrial fluid-blood barrier (review). Hear. Res..

[4] Hirose K, Li SZ, Ohlemiller KK, Ransohoff RM (2014). Systemic lipopolysaccharide induces cochlear inflammation and exacerbates the synergistic ototoxicity of kanamycin and furosemide. J. Assoc. Res. Otolaryngol. JARO.

[5] Reiss M, Reiss G (2009). Presbyacusis: Pathogenesis and treatment. Med. Monatsschr. Pharm..

[6] Monasta L, Ronfani L, Marchetti F, Montico M, Vecchi Brumatti L, Bavcar A, Grasso D, Barbiero C, Tamburlini G (2012). Burden of disease caused by otitis media: systematic review and global estimates. PloS one.

[7] Schilder AG, Chonmaitree T, Cripps AW, Rosenfeld RM, Casselbrant ML, Haggard MP, Venekamp RP (2016). Otitis media. Nat. Rev. Dis. Primers.

[8] Jiang H, Wu C, Xu J, Wang Q, Shen L, Ou X, Liu H, Han X, Wang J, Ding W, Hu L, Chen X (2021). Bacterial and fungal infections promote the bone erosion progression in acquired cholesteatoma revealed by metagenomic next-generation sequencing. Front. Microbiol..

[9] Kazmierczak W, Janiak-Kiszka J, Budzynska A, Nowaczewska M, Kazmierczak H, Gospodarek-Komkowska E (2022). Analysis of pathogens and antimicrobial treatment in different groups of patients with chronic otitis media. J. Laryngol. Otol..

[10] Chai Y, He W, Yang W, Hetrick AP, Gonzalez JG, Sargsyan L, Wu H, Jung TTK, Li H (2021). Intratympanic lipopolysaccharide elevates systemic fluorescent gentamicin uptake in the cochlea. Laryngoscope.

[11] Forge A, Schacht J (2000). Aminoglycoside antibiotics. Audiol. Neurootol..

[12] Dai CF, Mangiardi D, Cotanche DA, Steyger PS (2006). Uptake of fluorescent gentamicin by vertebrate sensory cells in vivo. Hear. Res..

[13] Di Pietro P, Dellacasaalberighi O, Silvestri M, Tosca MA, Ruocco A, Conforti G, Rossi GA, Castagnola E, Merlano MC, Zappettini S, Renna S (2017). Monitoring adherence to guidelines of antibiotic use in pediatric pneumonia: The MAREA study. Ital. J. Pediatrics.

[14] Khatun MR, Alam KMF, Naznin M, Salam MA (2021). Microbiology of chronic suppurative otitis media: An update from a tertiary care hospital in Bangladesh. Pak. J. Med. Sci..

[15] van Olst L, Roks SJM, Kamermans A, Verhaar BJH, van der Geest AM, Muller M, van der Flier WM, de Vries HE (2021). Contribution of gut microbiota to immunological changes in alzheimer's disease. Front. Immunol..

[16] Pu Q, Lin P, Gao P, Wang Z, Guo K, Qin S, Zhou C, Wang B, Wu E, Khan N, Xia Z, Wei X, Wu M (2021). Gut microbiota regulate gut-lung axis inflammatory responses by mediating ILC2 compartmental migration. J. Immunol..

[17] Ke S, Pollock NR, Wang XW, Chen X, Daugherty K, Lin Q, Xu H, Garey KW, Gonzales-Luna AJ, Kelly CP, Liu YY (2021). Integrating gut microbiome and host immune markers to understand the pathogenesis of Clostridioides difficile infection. Gut Microbes.

[18] Kociszewska D, Vlajkovic S (2022). Age-related hearing loss: The link between inflammaging, immunosenescence, and gut dysbiosis. Int. J. Mol. Sci..

[19] Juergens UR (2014). Anti-inflammatory properties of the monoterpene 1.8-cineole: Current evidence for co-medication in inflammatory airway diseases. Drug Res..

[20] Juergens UR, Stober M, Vetter H (1998). Inhibition of cytokine production and arachidonic acid metabolism by eucalyptol (1.8-cineole) in human blood monocytes in vitro. Eur. J. Med. Res..

[21] Ocana A, Reglero G (2012). Effects of thyme extract oils (from thymus vulgaris, thymus zygis, and thymus hyemalis) on cytokine production and gene expression of oxLDL-stimulated THP-1-macrophages. J. Obes..

[22] Juergens UR, Engelen T, Racke K, Stober M, Gillissen A, Vetter H (2004). Inhibitory activity of 1,8-cineol (eucalyptol) on cytokine production in cultured human lymphocytes and monocytes. Pulm. Pharmacol. Ther..

[23] MacKenzie C, Goerke T, Buecking M, Heidemann M, Leichtle A, Ringbeck B, Mollenkolk F, Ploch M, Bruchhage KL, Pries R (2023). Determination of orally administered 1,8-Cineol in nasal polyp tissues from chronic rhinosinusitis patients using gas chromatography: Mass spectrometry. Sci. Rep..

[24] Moo CL, Osman MA, Yang SK, Yap WS, Ismail S, Lim SH, Chong CM, Lai KS (2021). Antimicrobial activity and mode of action of 1,8-cineol against carbapenemase-producing *Klebsiella*
*pneumoniae*. Sci. Rep..

[25] Juergens UR, Dethlefsen U, Steinkamp G, Gillissen A, Repges R, Vetter H (2003). Anti-inflammatory activity of 1.8-cineol (eucalyptol) in bronchial asthma: A double-blind placebo-controlled trial. Respir. Med..

[26] Bruchhage KL, Koennecke M, Drenckhan M, Plotze-Martin K, Pries R, Wollenberg B (2018). 1,8-cineol inhibits the Wnt/beta-catenin signaling pathway through GSK-3 dephosphorylation in nasal polyps of chronic rhinosinusitis patients. Eur. J. Pharmacol..

[27] Schurmann M, Oppel F, Gottschalk M, Buker B, Jantos CA, Knabbe C, Hutten A, Kaltschmidt B, Kaltschmidt C, Sudhoff H (2019). The therapeutic effect of 1,8-cineol on pathogenic bacteria species present in chronic rhinosinusitis. Front. Microbiol..

[28] Sokovic M, Glamoclija J, Marin PD, Brkic D, van Griensven LJ (2010). Antibacterial effects of the essential oils of commonly consumed medicinal herbs using an in vitro model. Molecules.

[29] Ghavam M, Manca ML, Manconi M, Bacchetta G (2020). Chemical composition and antimicrobial activity of essential oils obtained from leaves and flowers of *Salvia*
*hydrangea* DC. ex Benth. Sci. Rep..

[30] Niedzielski A, Chmielik LP, Stankiewicz T (2021). The formation of biofilm and bacteriology in otitis media with effusion in children: A prospective cross-sectional study. Int. J. Environ. Res. Public Health.

[31] Daniel M, Imtiaz-Umer S, Fergie N, Birchall JP, Bayston R (2012). Bacterial involvement in otitis media with effusion. Int. J. Pediatric Otorhinolaryngol..

[32] Hall-Stoodley L, Stoodley P (2009). Evolving concepts in biofilm infections. Cell. Microbiol..

[33] Merghni A, Noumi E, Hadded O, Dridi N, Panwar H, Ceylan O, Mastouri M, Snoussi M (2018). Assessment of the antibiofilm and antiquorum sensing activities of *Eucalyptus*
*globulus* essential oil and its main component 1,8-cineole against methicillin-resistant Staphylococcus aureus strains. Microb. Pathog..

[34] Landeo-Villanueva GE, Salazar-Salvatierra ME, Ruiz-Quiroz JR, Zuta-Arriola N, Jarama-Soto B, Herrera-Calderon O, Pari-Olarte JB, Loyola-Gonzales E (2023). Inhibitory activity of essential oils of Mentha spicata and Eucalyptus globulus on biofilms of Streptococcus mutans in an in vitro model. Antibiotics.

[35] Addo KA, Li L, Li H, Yu Y, Xiao X (2022). Osmotic stress relief antibiotic tolerance of 1,8-cineole in biofilm persister cells of Escherichia coli O157:H7 and expression of toxin-antitoxin system genes. Microb. Pathog..

[36] Vazquez NM, Mariani F, Torres PS, Moreno S, Galvan EM (2020). Cell death and biomass reduction in biofilms of multidrug resistant extended spectrum beta-lactamase-producing uropathogenic Escherichia coli isolates by 1,8-cineole. PloS One.

[37] Hendry ER, Worthington T, Conway BR, Lambert PA (2009). Antimicrobial efficacy of eucalyptus oil and 1,8-cineole alone and in combination with chlorhexidine digluconate against microorganisms grown in planktonic and biofilm cultures. J. Antimicrob. Chemother..

[38] Annapoorani A, Jabbar AK, Musthafa SK, Pandian SK, Ravi AV (2012). Inhibition of quorum sensing mediated virulence factors production in urinary pathogen serratia marcescens PS1 by marine sponges. Indian J. Microbiol..

[39] LaSarre B, Federle MJ (2013). Exploiting quorum sensing to confuse bacterial pathogens. Microbiol. Mol. Biol. Rev. MMBR.

[40] Packiavathy IA, Priya S, Pandian SK, Ravi AV (2014). Inhibition of biofilm development of uropathogens by curcumin - an anti-quorum sensing agent from Curcuma longa. Food Chem..

[41] Li L, Li ZW, Yin ZQ, Wei Q, Jia RY, Zhou LJ, Xu J, Song X, Zhou Y, Du YH, Peng LC, Kang S, Yu W (2014). Antibacterial activity of leaf essential oil and its constituents from *Cinnamomum*
*longepaniculatum*. Int. J. Clin. Exp. Med..

[42] Kaltschmidt BP, Ennen I, Greiner JFW, Dietsch R, Patel A, Kaltschmidt B, Kaltschmidt C, Hutten A (2020). Preparation of terpenoid-invasomes with selective activity against *S*. *aureus* and characterization by cryo transmission electron microscopy. Biomedicines.

[43] Vijay A, Valdes AM (2022). Role of the gut microbiome in chronic diseases: A narrative review. Eur. J. Clin. Nutr..

[44] Arumugam M, Raes J, Pelletier E, Le Paslier D, Yamada T, Mende DR, Fernandes GR, Tap J, Bruls T, Batto JM, Bertalan M, Borruel N, Casellas F, Fernandez L, Gautier L, Hansen T, Hattori M, Hayashi T, Kleerebezem M, Kurokawa K, Leclerc M, Levenez F, Manichanh C, Nielsen HB, Nielsen T, Pons N, Poulain J, Qin J, Sicheritz-Ponten T, Tims S, Torrents D, Ugarte E, Zoetendal EG, Wang J, Guarner F, Pedersen O, de Vos WM, Brunak S, Dore J, Meta HITC, Antolin M, Artiguenave F, Blottiere HM, Almeida M, Brechot C, Cara C, Chervaux C, Cultrone A, Delorme C, Denariaz G, Dervyn R, Foerstner KU, Friss C, van de Guchte M, Guedon E, Haimet F, Huber W, van Hylckama-Vlieg J, Jamet A, Juste C, Kaci G, Knol J, Lakhdari O, Layec S, Le Roux K, Maguin E, Merieux A, Melo Minardi R, M'Rini C, Muller J, Oozeer R, Parkhill J, Renault P, Rescigno M, Sanchez N, Sunagawa S, Torrejon A, Turner K, Vandemeulebrouck G, Varela E, Winogradsky Y, Zeller G, Weissenbach J, Ehrlich SD, Bork P (2011). Enterotypes of the human gut microbiome. Nature.

[45] Wu M, Kasper DL (2020). Fiber sets up the battleground for intestinal prevotella. Cell Host Microbe.

[46] Agus A, Clement K, Sokol H (2021). Gut microbiota-derived metabolites as central regulators in metabolic disorders. Gut.

[47] Tajiri K, Shimizu Y (2018). Branched-chain amino acids in liver diseases. Transl. Gastroenterol. Hepatol..

[48] Roager HM, Licht TR, Poulsen SK, Larsen TM, Bahl MI (2014). Microbial enterotypes, inferred by the prevotella-to-bacteroides ratio, remained stable during a 6-month randomized controlled diet intervention with the new nordic diet. Appl. Environ. Microbiol..

[49] Lim GB (2020). Improved gut microbiota profile in individuals with obesity taking statins. Nat. Rev. Cardiol..

[50] Wolf EA, Rettig HC, Lupatsii M, Schluter B, Schafer K, Friedrich D, Graspeuntner S, Rupp J (2021). Culturomics approaches expand the diagnostic accuracy for sexually transmitted infections. Int. J. Mol. Sci..

[51] Graspeuntner S, Bohlmann MK, Gillmann K, Speer R, Kuenzel S, Mark H, Hoellen F, Lettau R, Griesinger G, Konig IR, Baines JF, Rupp J (2018). Microbiota-based analysis reveals specific bacterial traits and a novel strategy for the diagnosis of infectious infertility. PloS One.

[52] Graspeuntner S, Loeper N, Kunzel S, Baines JF, Rupp J (2018). Selection of validated hypervariable regions is crucial in 16S-based microbiota studies of the female genital tract. Sci. Rep..

[53] Schloss PD, Westcott SL, Ryabin T, Hall JR, Hartmann M, Hollister EB, Lesniewski RA, Oakley BB, Parks DH, Robinson CJ, Sahl JW, Stres B, Thallinger GG, Van Horn DJ, Weber CF (2009). Introducing mothur: Open-source, platform-independent, community-supported software for describing and comparing microbial communities. Appl. Environ. Microbiol..

[54] Parks DH, Chuvochina M, Waite DW, Rinke C, Skarshewski A, Chaumeil PA, Hugenholtz P (2018). A standardized bacterial taxonomy based on genome phylogeny substantially revises the tree of life. Nat. Biotechnol..

[55] McDonald D, Price MN, Goodrich J, Nawrocki EP, DeSantis TZ, Probst A, Andersen GL, Knight R, Hugenholtz P (2012). An improved Greengenes taxonomy with explicit ranks for ecological and evolutionary analyses of bacteria and archaea. ISME J..

[56] Rognes T, Flouri T, Nichols B, Quince C, Mahe F (2016). VSEARCH: A versatile open source tool for metagenomics. PeerJ.

[57] Afgan E, Baker D, Batut B, van den Beek M, Bouvier D, Cech M, Chilton J, Clements D, Coraor N, Gruning BA, Guerler A, Hillman-Jackson J, Hiltemann S, Jalili V, Rasche H, Soranzo N, Goecks J, Taylor J, Nekrutenko A, Blankenberg D (2018). The Galaxy platform for accessible, reproducible and collaborative biomedical analyses: 2018 update. Nucleic Acids Res..

[58] Segata N, Izard J, Waldron L, Gevers D, Miropolsky L, Garrett WS, Huttenhower C (2011). Metagenomic biomarker discovery and explanation. Genome Biol..

