# Supplementary material for: Anti-inflammatory response to 1,8-Cineol and associated microbial communities in Otitis media patients
Source: Sci Rep. 2024 Jul 16;14:16362. doi: 10.1038/s41598-024-67498-5 (PMC11252366; doi:10.1038/s41598-024-67498-5)
Supplement: Supplementary file 1 — Supplementary Information. [file 41598_2024_67498_MOESM1_ESM.pdf]

Supplement

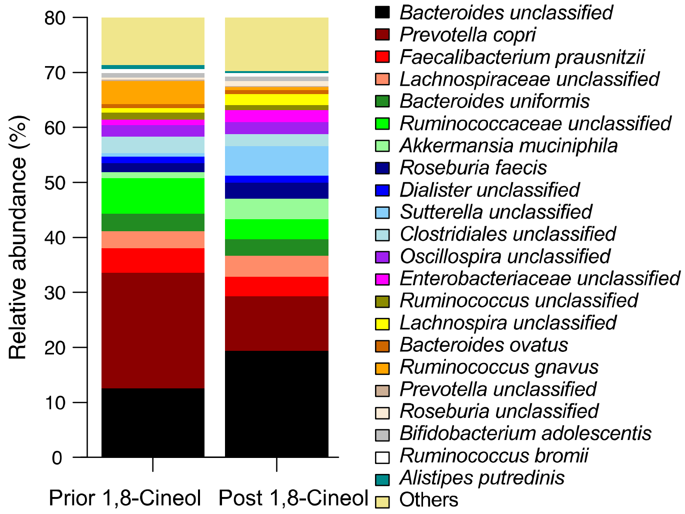

Figure 1S. Comparison of gut microbiota from COM patients before and after 14 days of 1,8-Cineol administration (3 times per day 200 mg Soledum forte). Depicted are relative abundances of 22 operational taxonomic units listed in the descending order.

Table 1S: Clinical and demographic information from patients utilized in this study. (N=no and Y=yes)

Patient	Gender	Age (years)	Otorrhea	Hearing Loss	Vertigo	Primary Diagnosis/Surgery	Secondary diagnosis
1	M	70	Y	Y	N	COM / Tymp-III in 2018	CRS, Smoker
2	F	36	Y	Y	Y	COM with mastoiditis /Tymp, Mastoidektomie and multiple T-Tubes	GPA, CRS, Smoker
3	M	49	Y	Y	N	COM, chronic tube dysfunction, /multiple ventilation Tubes	CRwNP, Asthma bronchiale
4	M	56	Y	N	N	COM, chronic tube dysfunction, /inserted vent Tube	Psoriasis (MTX-treatment), CRS
5	F	68	Y	Y	N	Recurrent COM	Diabetes, COPD, CRS, Smoker
6	M	53	Y	Y	N	Granulating COM	suspected GPA, CRS, Smoker
7	M	30	Y	Y	Y	COM with vertigo / multiple Tymp-III in childhood, multiple vent Tubes	Asthma bronchiale, CRS
8	M	67	Y	Y	N	Granulating COM with Mastoiditis	CRS
9	M	55	Y	Y	N	Persistent COM	Diabetes, COPD, CRS
10	F	44	Y	Y	N	Recurrent COM	CRS
